# Optically Detected Magnetic Resonance Based Intracellular Thermometry Using Nanodiamonds Implanted in Adherent Cancer Cells

**DOI:** 10.1021/acsanm.5c05655

**Published:** 2026-02-15

**Authors:** John C. Consiglio, Rostislav Boltyanskiy, Yuliya L. Mindarava, Christian Laube, Wolfgang Knolle, Marjan Berishaj, Fedor Jelezko, Kayvan R. Keshari

**Affiliations:** † Center for Molecular Imaging and Bioengineering, 5803 Memorial Sloan Kettering Cancer Center, New York, New York 10065, United States; ‡ Department of Functional Surfaces, Leibniz Institute of Surface Engineering, Leipzig 04318, Germany; § Institute for Quantum Optics, 9189 Ulm University, 89081 Ulm, Germany; ∥ Department of Radiology, 5803 Memorial Sloan Kettering Cancer Center, New York, New York 10065, United States; ⊥ Molecular Pharmacology Program, 5803 Memorial Sloan Kettering Cancer Center, New York, New York 10065, United States; # Center for Integrated Quantum Science and Technology (IQST), 9189 Ulm University, 89081 Ulm, Germany

**Keywords:** Intracellular thermometry, nanodiamonds, NV-center, glioblastoma, metabolism, optically detected magnetic resonance, quantum sensing

## Abstract

Intracellular temperature influences cell metabolism and the tumor microenvironment, but there is a lack of consensus on basic thermal properties of cells and a shortage of reliable measurement tools. We utilized nanodiamonds (NDs) containing fluorescent Nitrogen Vacancy centers (NVs) implanted in glioblastoma cells to measure intracellular temperature via Optically Detected Magnetic Resonance (ODMR) and used dual fluorescence and bright-field imaging to establish ND–mitochondria distances. Careful ND selection, improved curve fitting, and time averaging enable repeatable measurement of cellular responses. Upon mitochondrial uncoupling with 5 μM FCCP, we reproducibly observed ODMR shifts consistent with an ∼1.7 °C temperature increase.

## Graphical abstract

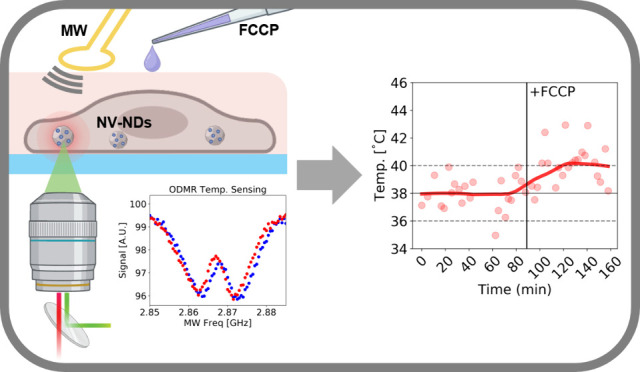

## Introduction

Temperature plays an important role in many vital cell functions including growth, metabolism, protein folding, DNA replication, and overall cell signaling.^1^ In addition to normal cell growth and development, altered cellular response to temperature changes have been implicated in various pathologies, including cancer.^2^ Given the high metabolic demand, the replicative immortality and other cancer hallmarks in many malignancies^3^ the role of temperature in the context of cancer is of particular interest. On the treatment side as well, hyperthermia therapy has been used to treat various cancers either as a stand-alone procedure^4^ or in combination with other treatments.^5^ Recently the tumor and overall body temperature have been monitored in response to cancer treatment as a preclinical proxy of immunotherapy and chemotherapy efficacy.^6^

While the role of temperature on a tissue-scale is universally recognized, on a single cell or intracellular scale, the science of temperature regulation and response to treatment is far less clear. Maximum possible intracellular heat generation and thermal gradients remain a subject of debate.^7^ One of the controversies at the root of the intracellular temperature debate is whether sufficient heat can be generated within single cells to raise the temperature by an order of 1 °C. While several groups have measured temperature changes on that scale,^8,9^ other groups have argued that such changes are not physically possible given the known energetics and thermal properties of cells.^10^ This debate is particularly relevant in the context of cancer, given the extraordinary heterogeneity of the tumor microenvironment.

To properly address the role of intracellular temperature, it is essential to utilize accurate, reliable tools. While the number of intracellular sensors has grown vastly in recent years, only a few are built for temperature sensing. Among the most common tools for measuring intracellular temperature are fluorescent polymers,^9^ organic dyes,^11^ Raman spectroscopy,^12^ and various fluorescent nanoparticles.^13^ Among fluorescent nanoparticles, nanodiamonds (NDs) containing negatively charged fluorescent Nitrogen Vacancy (NV^–^) centers offer a number of unique advantages.^14^ NDs are inert and are easily incorporated into cells.^15^ They exhibit superb photostability and robust sensitivity to numerous biophysical stimuli, particularly temperature.^16^ Several groups have utilized microdiamonds and NDs for temperature measurements of subcellular components,^17^ in cells^18,19^ and specifically in several cancer cell lines.^20,21^ However, there is limited ND measurement based data available related to the effect of metabolic perturbations on intracellular temperature^22,23^ and none available specifically for cancer cells.

In this work, we developed a robust analysis protocol to measure intracellular temperature including control experiments such as cell culture media exchange, addition of solvents (DMSO), and environmental pH changes. We then investigated the effect of a mitochondrial uncoupler, FCCP, on intracellular temperature by interrogating NV nanodiamonds internalized by U251 glioblastoma cells. Using bright-field and fluorescence imaging, we monitored and localized nanodiamonds with respect to subcellular components and compared changes in oxygen consumption rate to changes in cell temperature in response to the administration of 5 μM of FCCP.

Our temperature measurement technique utilized custom nanodiamonds containing NV^–^ centers. The NV^–^ center is a diamond crystal lattice defect consisting of a single substitutional nitrogen nucleus with an adjacent lattice vacancy forming a two-electron spin system, schematically represented in Figure 1A. The NV^–^ center is characterized by a spin triplet ground state exhibiting spin-state dependent fluorescence.^24^ Under excitation from a 532 nm laser the m_s_ = 0 sublevel is long cycling and exhibits a steady fluorescence whereas the m_s_= ±1 spin states show strong shelving into metastable singlet states, thereby reducing fluorescence intensity. The transition energy between the m_s_ = 0 (bright) and m_s_= ±1 (dim) states is termed zero field splitting, denoted D_gs_, and is in the microwave range around 2.87 GHz. The temperature dependence of D_gs_ is the basis of NV-based temperature sensing.

**Figure 1:**
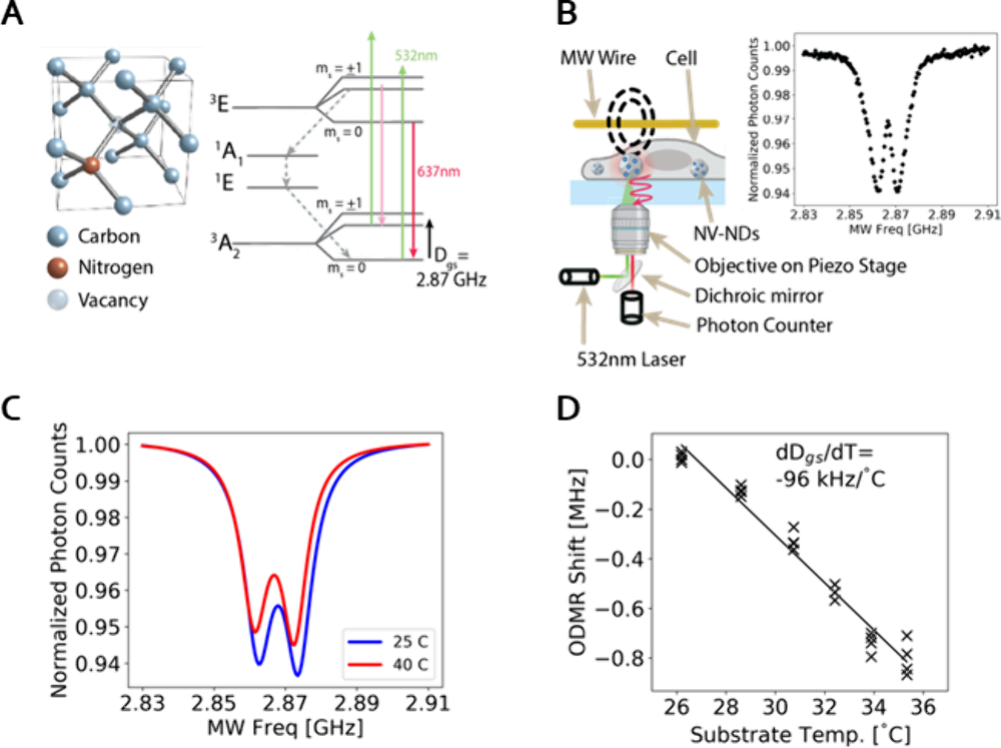
Using NV-nanodiamonds (NDs) for real-time temperature measurements. (A) Schematic showing the Nitrogen Vacancy defect in a diamond crystal on the left and the NV^–^ energy level diagram on the right. The m_s_ = 0 and m_s_ = ±1 spin states are shown in the ground state at the bottom and the excited state at the top. Laser excitation is indicated by green arrows, and emission by red arrows. The dimmer red arrow between the excited and ground m_s_ = ±1 spin states signifies reduced fluorescence. The energy gap that is most sensitive to temperature is indicated by the black arrow at the bottom of the energy diagram. (B) A schematic of the basic setup for the Optically Detected Magnetic Resonance (ODMR) experiment on the left and an example ODMR curve from which the m_s_= ±1 transition energies can be determined on the right. The ODMR curve is normalized by the maximum signal and is an average of 136 frequency sweeps. Created with BioRender.com. (C) An example ODMR curve from a single ND at 40 °C in red and 25 °C in blue. Each curve is a double Lorentzian fit to the ODMR data. (D) A calibration curve for a single ND on a glass slide resulting from stepwise adjustment of the slide temperature and simultaneous measurement of the ODMR peak shift. The data represents an example ND measured 4 times at each temperature.

To implement NV-ND based temperature measurements, we built a custom confocal microscope (Supporting Information, Figure S1). Figure 1B shows the critical components of the microscope, which allows for tracking of individual intracellular NDs while measuring fluorescence and irradiating with microwave energy. The inset of Figure 1B shows an example of normalized fluorescence intensity as a function of the microwave frequency in the vicinity of D_gs_. The two dips in the fluorescence intensity correspond to the m_s_ = +1 and m_s_ = −1 transitions. This optical detection of the spin state transitions is called optically detected magnetic resonance (ODMR) (Supporting Information, Figure S2). The value of D_gs_ is the arithmetic mean of the m_s_ = +1 and m_s_ = −1 frequencies and shifts in D_gs_ are calibrated to changes in temperature.

An example of a temperature-induced change in the ODMR curve is shown in Figure 1C, the results of an experiment in which a single ND was adhered to a glass slide and exposed to a water bath at 25 °C (in blue) and at 45 °C (in red). While contrast and line width may also vary, the change in ODMR center frequency, D_gs_, is most consistently associated with temperature change. Figure 1D shows an example calibration of the ODMR shift as a function of temperature for one of our NDs (ND preparation detailed in the Supporting Information: Methods). Calibration curves were obtained for several additional NDs (Table S1) resulting in an average sensitivity value of dD_gs_/dT = −91 ± 5 kHz/°C which was applied to all subsequent measurements.

To deploy NV-NDs as intracellular nanotemperature sensors, we developed a protocol to implant NDs in U251 glioblastoma cells and explored where they localize. The ND concentration and implantation processes were optimized to yield between 5 and 10 NDs incorporated in each cell (see Supporting Information: Methods).

To visualize ND dynamics in a cell and to explore where NDs localize, U251 cells were stained with LysoTracker Green and MitoTracker DeepRed. This staining allowed sequential imaging of cell morphology in bright-field, as well as imaging of NDs, lysosomes, and mitochondria in 3 different nonoverlapping fluorescent channels. Figure 2A shows an example bright-field image of several U251 cells and the NDs they engulfed. While NDs are found throughout the cell, they often accumulate in high number densities in specific regions of the cell. Figure 2B shows the same bright-field image but with the LysoTracker Green fluorescence channel showing lysosome locations. Figure 2C shows the same bright-field image again but now with the MitoTracker Red fluorescence channel showing the mitochondrial density. Figure 2D shows calculated ND centroids plotted as red dots and lysosome centroids plotted as empty green circles overlaid on both the bright-field and the mitochondrial density images. The majority of the red dots are contained within green circles (suggesting lysosomal engulfment) and in close proximity to mitochondria.

**Figure 2:**
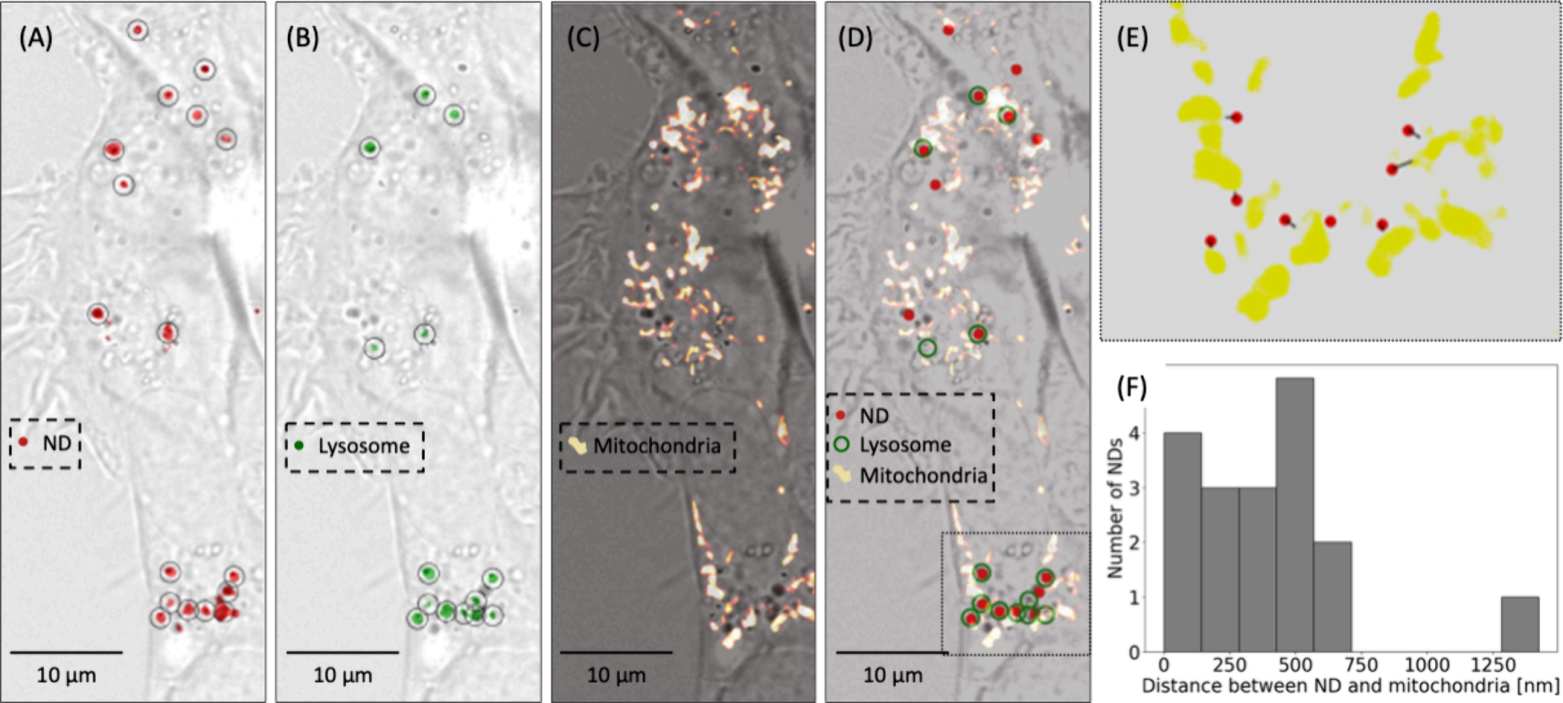
Implanting NDs in live cells. (A) Bright-field imaging of U251 cells containing NDs overlaid with a fluorescence image of engulfed NDs in red. Black circles are centered on automatically identified NDs. (B) Same bright-field image as in (A) but overlaid with a fluorescence image of LysoTracker Green dye taken up by cells, indicating Lysosomes location. Black circles are centered on automatically identified stained lysosomes. (C) Same bright-field image as in (A) but overlaid with a fluorescence image of MitoTracker Deep Red dye taken up by cells, indicating location of mitochondria. (D) Same bright-field image as in (A) with red dots indicating the centroids of engulfed NDs (from panel A), green circles indicating the centroids of lysosomes (from panel B) and the same mitochondrial fluorescence map as in panel C. (E) A close-up of a region delineated by the dotted lines in panel D with mitochondria in yellow, ND centroids as red dots, and lines connecting NDs to the nearest mitochondrial surface. (F) A histogram of distances in the X,Y plane between the NDs shown in panels A and D and the nearest mitochondria. This imaging was performed on a confocal microscope with a slice thickness of less than 1 μm, setting an upper limit on the ND to mitochondria separation in the Z dimension.

Using the ND centroids and a masked image of the mitochondrial density, we found the distance between each ND and the nearest mitochondrial surface. Figure 2E shows a section of Figure 2D with ND centroids connected to the nearest edge of a mitochondrial aggregate. Figure 2F shows a histogram of the distances between the 18 NDs shown in Figure 2E and the closest mitochondria. We note that NDs are adjacent to rather than inside of mitochondria with the majority of NDs less than 1 μm from the nearest mitochondrial surface.

While Figure 2 shows a static snapshot of NDs engulfed by lysosomes near mitochondria in an adherent cell, reality is dynamic. The cells migrate along the glass surface, NDs in lysosomes move around in a cell and the mitochondria rearrange in density waves. A video capturing these dynamics is included in the Supporting Information.

To ensure robustness and reliability of the intracellular ND temperature sensor, we performed a number of control experiments. During the course of the experiment, before the addition of FCCP, we refilled the PDMS well with 100 μL of heated media to ensure a sufficient nutrient supply and an environmental steady state for the cells. To explore if the media addition affects apparent cell temperature, we measured intracellular temperature with an internalized ND, before and after media addition, in 4 different cells (Figure 3A). In each experiment all temperature values are normalized to the average value of the prerefill measurements, which were collected continuously for a minimum of 20 min before addition of media. Results are presented as temperature changes relative to the prerefill baseline. Distinct markers represent NDs in different cells acquired on different days. A paired *t* test shows no significant change in intracellular temperature due to refilling of the media.

**Figure 3:**
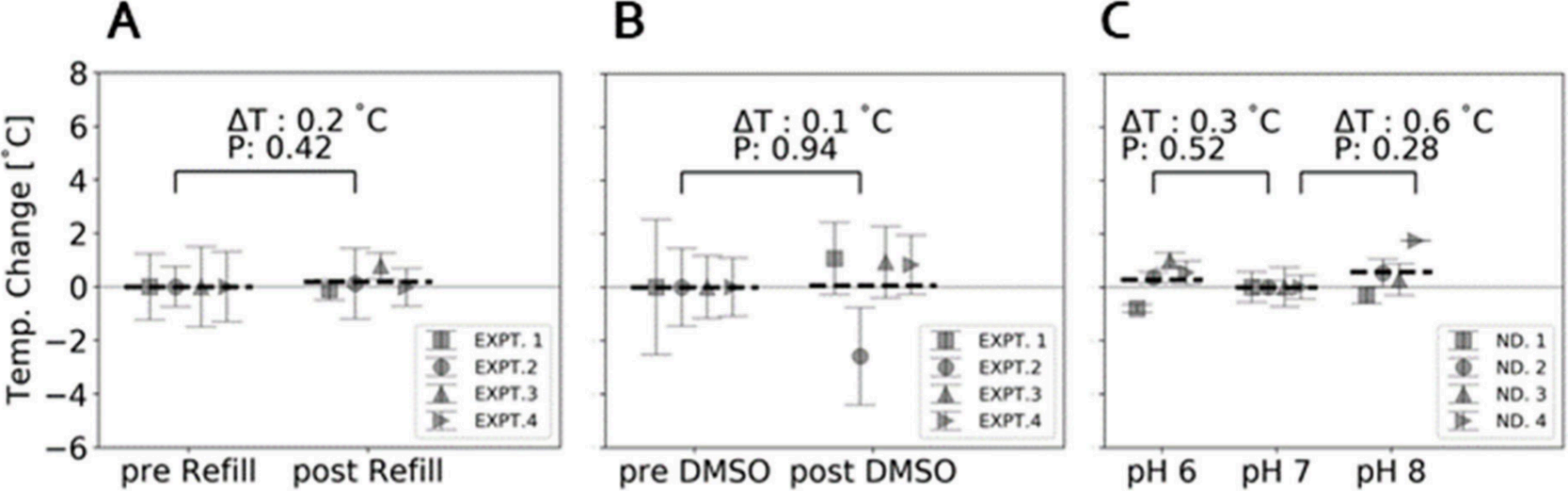
Control Experiments. (A) Comparison of intracellular ND reported temperature sampled for approximately 20 min before and after adding new media to the temperature regulated cell incubation chamber. Each marker represents one ND in one cell. Different markers are NDs from different cells tested on different days. Dashed black horizontal line represents the average temperature across the four experimental repeats. (B) Same as (A) but instead of exchanging media, 0.125 μL of DMSO was added to the temperature regulated cell incubation chamber. Data is a compilation of 4 experimental repeats as in (A). (C) Comparison of temperature reported by NDs adhered directly on the glass slide exposed to three different pH buffer solutions in the temperature regulated cell incubation chamber. The pH buffer solution was exchanged by triple washing. Each marker is a separate ND but adhered to the same glass coverslip.

It was also important to check the effect of DMSO on cell temperature as our metabolic drug, FCCP, was dissolved in DMSO. For 20 min before and 40 min after addition of 0.125 μL of DMSO to the 500 μL chamber, an intracellular nanodiamond was tracked and its temperature was measured. As in Figure 3A, in Figure 3B the change in temperature is reported with distinct markers representing NDs in different cells each used for a separate experiment. Although several post-DMSO temperature readings showed either elevated or reduced values with respect to the baseline average of pre-DMSO values, a paired *t* test applied to the 4 interrogated NDs showed no significant temperature change.

Numerous phenomena, including administration of various drugs can affect intracellular pH.^25^ To assess ND sensitivity to pH changes, we interrogated 4 NDs adhered to a glass slide in a temperature-controlled bath and exposed them to pH buffers of pH 6, 7, and 8 (Figure 3C). The temperatures of the 4 NDs were measured continuously for 30 min at pH 7 followed 30 min at pH 6 and 30 min at pH 8. Figure 3C shows the measured change in temperature as a function of pH buffer, normalized to the temperature measurements at pH 7. A paired *t* test shows no significant changes in temperature readings for the raised and lowered pH values.

To observe the effect of a known metabolic drug that is often implicated in heat generation,^26^ we administered a 5 μM dose of FCCP to U251 cells. FCCP acts to disrupt the proton gradient across the inner mitochondrial membrane which reduces ATP synthesis^27^ (Figure 4A). This reduction in ATP production spurs an increase in the rate of oxidative phosphorylation and a rise in the oxygen consumption rate.

**Figure 4:**
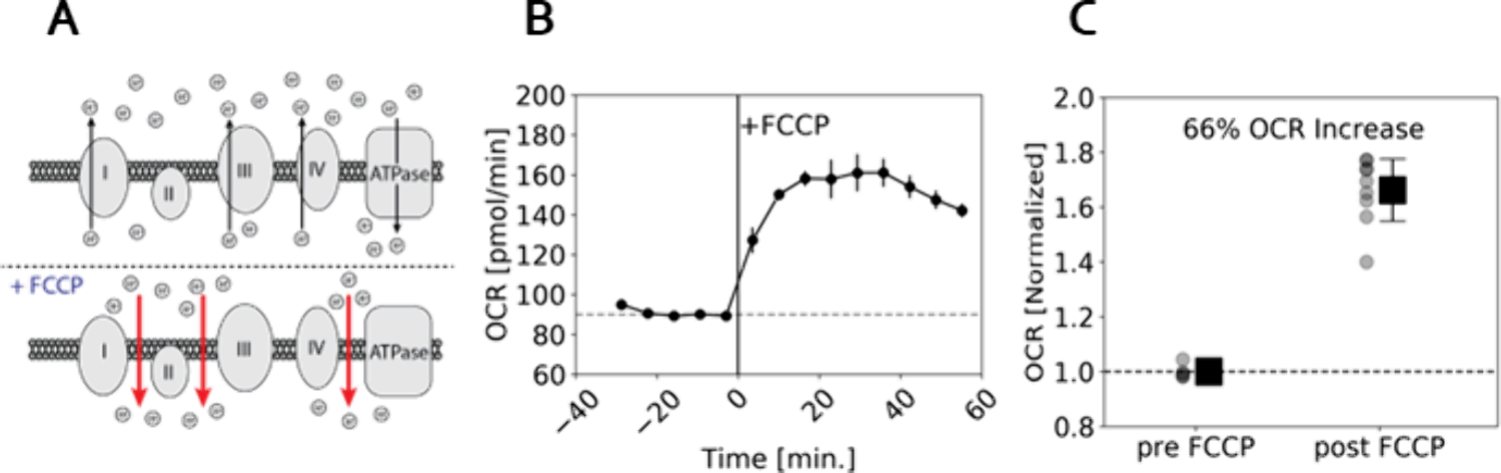
Increased metabolic activity following FCCP administration. (A) A schematic of the inner mitochondrial membrane showing the three protein complexes that work to create an H+ gradient across the membrane and the ATP synthase which uses the H+ gradient in the synthesis of ATP. The lower panel shows the effect of the mitochondrial uncoupler, FCCP, which make the membrane porous and allows the H+ gradient to quickly dissipate across the membrane, reducing ATP Synthesis. (B) Results of Extracellular Flux measurement of the Oxygen Consumption Rate (OCR) before and after addition of 5 μM FCCP to a population of 20,000 U251 cells. Each point is a measurement that averages the OCR of 20,000 cells across 12 different wells. (C) Normalized data from (B) comparing all pre- and post FCCP OCR measurements showing 66% (SD = 11%) OCR increase upon addition of FCCP. The positions of the black boxes show the medians of the populations, and the error bars represent one standard deviation.

To determine an optimal FCCP dose, we used the Seahorse instrument to measure the oxygen consumption rate (OCR) of a population of adherent U251 cells during administration of a range of FCCP concentrations. Figure 4B shows the increase in the level of the OCR in response to a 5 μM FCCP dose. Supporting Information Figure S4 shows OCR response curves of the OCR to other FCCP concentrations. Each point in Figure 4B corresponds to an OCR of approximately 20,000 cells, averaged over 12 wells. A sharp rise in the level of the OCR is reproducibly observed within 20 min of FCCP administration. A normalized comparison of OCR pre- and post-FCCP administration shows a 66% (SD = 11%) increase in OCR (Figure 4C).

Next, we measured cell temperature response to administration of FCCP by incubating U251 cells with a dilute suspension of NDs and then mounting the cell incubation chamber in our custom microscope (Supporting Information Figure S1B). The ND containing cells were then imaged with the widefield imaging system to identify regions with healthy cells, followed by confocal fluorescence imaging to identify individual NDs which are suitable for temperature measurement (Supporting Information Table S2 for ND selection criteria). A single ND was then chosen and tracked throughout the experiment, and temperature was measured for 60–90 min before a 10 μL dose of FCCP solution was added to the media-filled incubation chamber (for a final concentration of 5 μM). Figure 5A shows an example temperature response curve from this experiment. The vertical black line represents the administration of FCCP at 89 min. While temperature measurements fluctuate significantly, there is a robust rise of up to several degrees that starts 10–15 min after FCCP addition. The temperature stayed elevated during the next 1 h of measurements.

**Figure 5:**
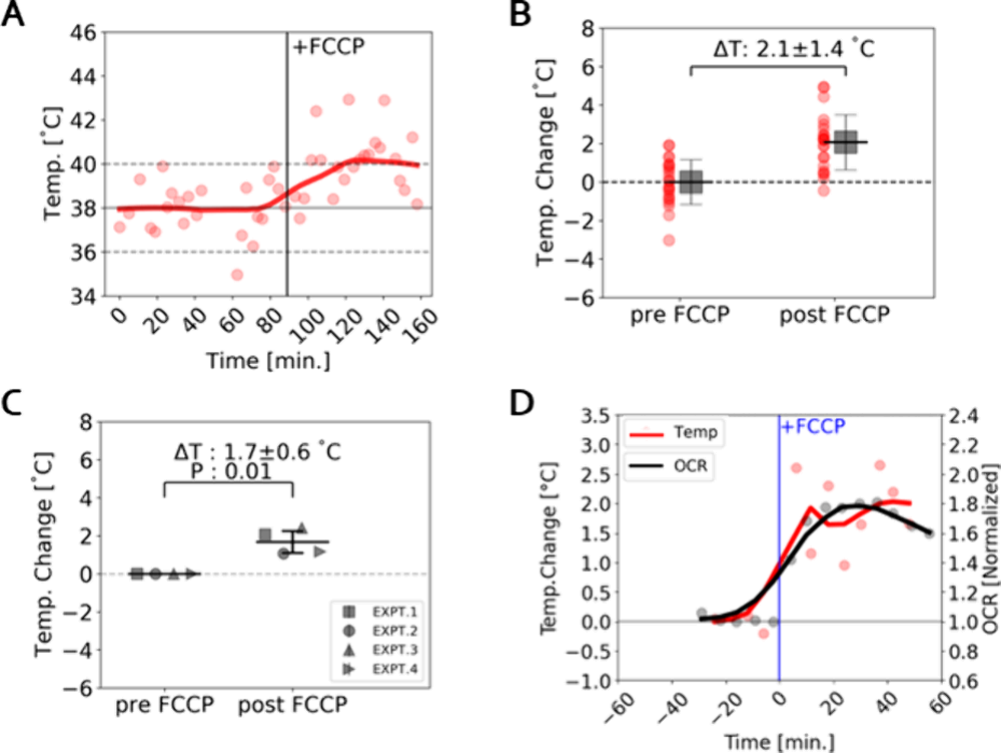
Effect of metabolic perturbation on intracellular temperature. (A) Temperature reported by a single intracellular ND for 89 min before and 66 min after administration of 5 μM FCCP. Each data point represents a temperature measurement extracted from an average of 136 ODMR curves acquired over 150 s. All temperature values are offset by a common normalizing constant to set the average of the first 10 data points to 38 °C. Trend line created by performing Locally Weighted Scatterplot Smoothing (LOWESS). (B) Comparison of intracellular temperature measurements before (23 points) and after (22 points) FCCP administration compiled from (A) and with the average of pre-FCCP measurements normalized to zero to show temperature change (2.1 ±1.4 °C, *p* = 0.00001). Low p-value indicates ability to use dense time sampling to detect a significant change despite a noisy measurement. (C) Comparison of pre and post FCCP temperature for four experimental repeats, including data from (B) labeled as EXPT.1. Each marker represents temperature measurements from 1 ND in 1 cell treated with 5 μM FCCP and performed on a different day (1.7 ± 0.6 °C, *p* = 0.01). (D) Overlay of temperature, in red, and OCR response, in black, to FCCP administration. The red dots represent an average of 4 experiments with different intracellular NDs in different cells. Data was binned into 6 min intervals and averaged together. Black dots represent average OCR as in Figure 4B. Trend lines are created by performing Locally Weighted Scatterplot Smoothing (LOWESS) of the corresponding, color-coded, data.

## Conclusion

Investigating the temperature change after FCCP administration for this example experiment (Figure 5B) we find a temperature rise of 2.1 ±1.4 °C. Moreover, when comparing 4 biological replicates, intracellular temperature readings are 1.7 ± 0.6 °C higher after addition of FCCP than before (Figure 5C, p = 0.01). Comparing the average temperature response of the 4 biological replicates to the OCR measurements in time provides a direct comparison of temporal dynamics with response to FCCP administration (Figure 5D). We observe a correspondence between the timing of maximum temperature increase (32 ± 5 min) and maximum OCR increase (32 ± 5 min), consistent with the notion that mitochondrial uncoupling correlates with increased temperature.

Our results indicate that intracellular temperature changes can be measured using nanodiamond-based thermometry but that large measurement variability necessitates a statistical treatment of the temperature data. Recent work has suggested that complex intracellular electric field changes can affect ND based temperature measurements, and that functionalized NDs and alternative measurement techniques can reduce these effects.^22^ In our work we seek to overcome these challenges by employing a careful ND characterization and selection process, performing biological control experiments, and acquiring larger data sets which can be analyzed statistically. We view our approach as complementary to recently published studies in this field^22^ while also noting important differences in cell type and sensor mitochondrial proximity. Additional discussion of nonthermal influences on ODMR shape parameters is presented in Supporting Information: OMDR Shape Parameters during the FCCP and Control Experiments.

Using our data collection and analysis techniques, we observe changes in ODMR center frequency consistent with an intracellular temperature increase of 1.7 °C in response to metabolic perturbation via FCCP which is in line with previously reported results. Using NDs and other techniques, other groups reported temperature increases of 1.02 °C in COS-7 Fibroblast-like cells,^9^ 1.60 °C in Brown Adipose Cells^28^ and 4.0 °C in C-Elegans worms^29^ following FCCP administration. While these experimental results are convincing, theoretical modeling predicts that the maximum intracellular temperature increase could not surpass 1e-5 °C based on presumed values of intracellular thermal properties.^10^ It is possible that thermal properties of cells are vastly miscalculated, and it has also been suggested that higher than expected levels of heat generation and unaccounted for heat transfer render existing modeling insufficient.^30^ Furthermore, our current work is with a cancer cell model that may exhibit a more aggressive metabolism than previously explored in the context of cell thermometry. Finally, beyond heat generated in mitochondria, drug-induced signaling may cause a cascade of exothermic reactions that can raise the cell temperature by generating heat in other cell compartments.

In conclusion, we developed a method to use NV^–^ nanodiamonds (NDs) for intracellular thermometry and applied it to the study of temperature changes in U251 glioblastoma cells. By identifying a protocol to extract temperature information from ODMR data and focusing on the consistency and reproducibility of our results, we strengthen the argument for a thermal cause of ODMR shifts. Using this approach, we observed temperature changes on the order of 1.7 °C following FCCP administration, further supporting the idea that bulk cellular temperature changes can be linked to biological processes. In future work we aim to further our understanding of the connection between metabolism and intracellular thermodynamics by studying additional cell types, improving our NV-ND sensor, and incorporating other metabolic perturbations.

## Supplementary Material





## Notes

Additional details, system characterization, ODMR shape parameters, and methods (PDF)
Nanodiamonds near mitochondria (AVI)

## Abbreviations

ND: nanodiamond
NV: Nitrogen Vacancy
ODMR: Optically Detected Magnetic Resonance
FCCP: Carbonyl cyanide 4-(trifluoromethoxy)­phenylhydrazone
DMSO: Dimethyl sulfoxide
PDMS: Polydimethylsiloxane
OCR: Oxygen Consumption Rate
fwhm: Full width at half-maximum.
