# Supplementary material for: Optically Detected Magnetic Resonance Based Intracellular Thermometry Using Nanodiamonds Implanted in Adherent Cancer Cells
Source: ACS Appl Nano Mater. 2026 Feb 15;9(8):3590–6. doi: 10.1021/acsanm.5c05655 (PMC12954747; doi:10.1021/acsanm.5c05655)
Supplement: Supplementary file 1 [file an5c05655_si_001.pdf]

Supporting Information, Optically detected magnetic resonance based intracellular thermometry using nanodiamonds implanted in adherent cancer cells

John C. Consiglio[1], Rostislav Boltyanskiy[1], Yuliya L. Mindarava[3], Christian Laube[2], Wolfgang Knolle[2], Marjan Berishaj[4,5], Fedor Jelezko[3,6], Kayvan R. Keshari[1,4,5]*

[1]Center for Molecular Imaging and Bioengineering, Memorial Sloan Kettering Cancer Center, New York, NY 10065, USA

[2]Department of Functional Surfaces, Leibniz Institute of Surface Engineering, Leipzig, 04318, Germany

[3]Institute for Quantum Optics, Ulm University, 89081 Ulm, Germany

[4]Department of Radiology, Memorial Sloan Kettering Cancer Center, New York, NY 10065, USA

[5]Molecular Pharmacology Program, Memorial Sloan Kettering Cancer Center, New York, NY 10065, USA

[6]Center for Integrated Quantum Science and Technology (IQST), Ulm University, 89081 Ulm, Germany

Correspondence*: rahimikk@mskcc.org (KRK)

Contents

Custom dual path quantum microscope S3
NV-ND based temperature measurement.. S5
Localization of intracellular NDs S10
Selecting FCCP Concentration.. S10
ODMR Shape Parameters during FCCP and Control Experiments.. S11
Methods.. S15

Custom dual path quantum microscope

To visualize the adherent U251 glioblastoma cells and localize the engulfed NDs while measuring their intracellular temperature changes, we built a custom dual imaging confocal microscope with a widefield path. Cells were incubated in a custom-made chamber (Figure S1A) overnight to fully adhere and reach steady state. Cells were imaged with a heated 60x (NA 1.35) oil immersion objective mounted on an XYZ piezo stage (shown in Figure S1B). After illumination with a 532nm excitation laser the emitted fluorescence was reflected by a 45° mirror and directed towards the photon counter. With the flip mirror in the vertical position, emission light was uninhibited and directed, through various optics, into the photon counter (see Methods for details). To study cell morphology and proximity to the microwave wire, the 45° mirror was flipped into the horizontal position and a white LED light was turned on from above. This engaged the widefield arm of the microscope and allowed for transmission imaging of the cells. With this system we tracked cell health over several hours, collected widefield and fluorescent images, identified and tracked individual intracellular NDs and measured intracellular temperature.

Figure S1C shows example widefield and fluorescence images of a single adherent U251 cell. The fluorescent image includes a limited amount of white light that was allowed to pass in order to visualize the morphology of the cell on the same image as the fluorescent NDs. The fluorescence image with a bit of white light was made by scanning with the piezo stage and acquiring with the photon counter. On the left side of both the fluorescent and the widefield images a shadow of the 20μm gold microwire can be seen. Some of the nanodiamonds are visible both in fluorescence and in transmission while others only in fluorescence. Overall, the dual

imaging paths allowed for selection of cells with healthy morphologies and identification of NDs that can be tracked for temperature sensing.

Figure S1.

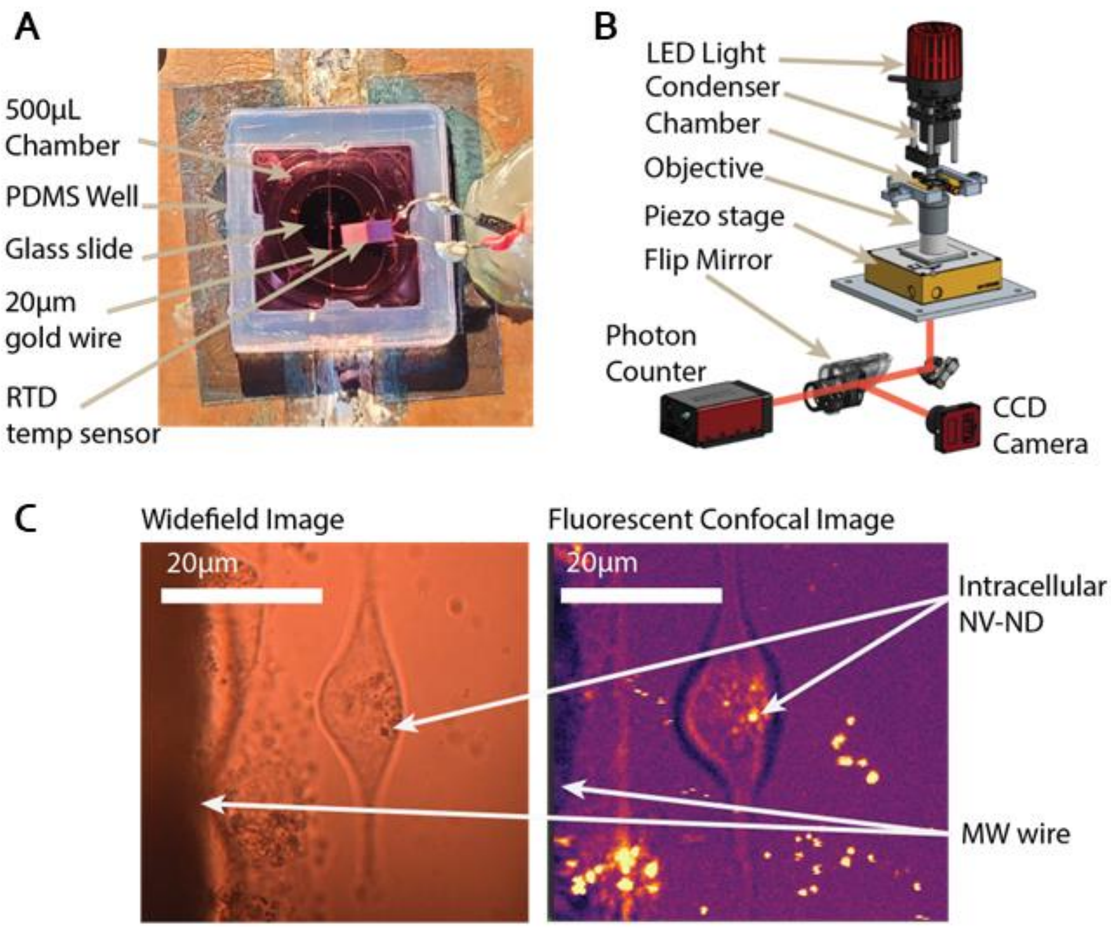

Figure S1. Dual path imaging for intracellular thermometry. (A) An image of a custom-made cell incubation chamber with a gold microwire in the middle and an RTD temperature sensor in an attached PDMS chamber filled with media. (B) Schematic of combined widefield and confocal microscope with custom cell incubation chamber. Schematic illustrates the use of a flip mirror to switch between the fluorescence path and the brightfield path. (C) A widefield transmission image of a U251 adherent glioblastoma cell on the left and a fluorescent scan with a limited amount of white light on the right. Arrows point to recognizable features in both images.

NV-ND based temperature measurement

The ODMR raw data that we collected consisted of a measurement of photon count frequency at evenly spaced intervals of microwave excitation. In our temperature measurement we excited microwave frequencies from 2.838 GHz to 2.898 GHz with a frequency step size of 1 MHz and 50ms MW excitation time at each step. In the frequency range near the ODMR peaks (2.858 GHz to 2.877 GHz) we used a reduced frequency step size of 0.5 MHz to improve the fitting function. For our temperature measurement each temperature point is extracted from averaging 136 ODMR curves acquired over a period of 150 seconds.

During initial temperature measurement trials, we fit the ODMR raw data using a double Lorentzian model (Figure S2A). This fitting provides two peak values, illustrated by the vertical dotted lines in Figure S2A, and a resulting center frequency, D_{gs}, was then calculated as the mean of the two peak values, shown as a solid vertical line. The fit uncertainty of the Double Lorentzian peak values (U_{peak1}, U_{peak2}) are propagated to find uncertainty values for the measured temperature (U_{Temp}) as shown in Equation S1 below.

$$U_{Temp} = \frac{\frac{1}{2}\sqrt{{U_{peak1}}^2 + {U_{peak2}}^2}}{\frac{dD_{gs}}{dt}}$$

Equation S1. Uncertainty propagation for finding temperature uncertainty from the uncertainty values of the ODMR peak fitting and the temperature sensitivity value.

Subsequently we developed an improved peak fitting method consisting of splitting the ODMR raw data at the center value between the two peaks and then fitting a single Lorentzian to

each of the two resulting data sets (Figure S2B). The center frequency, D_{gs}, is then calculated as the average of these two peaks values and the fit uncertainty of the two Single Lorentzian peak values are propagated to find uncertainty values for the measured temperature (also using Equation S1).

Figure S1C shows calculated temperature uncertainties based on these two fitting methods for 29 consecutive intracellular temperature measurements taken in a live U251 Glioblastoma cell over the course of 90 minutes. The two Single Lorentzian Fits method repeatably achieves a better fit to the ODMR data and was used in all intracellular temperature measurements reported in this work.

Figure S2.

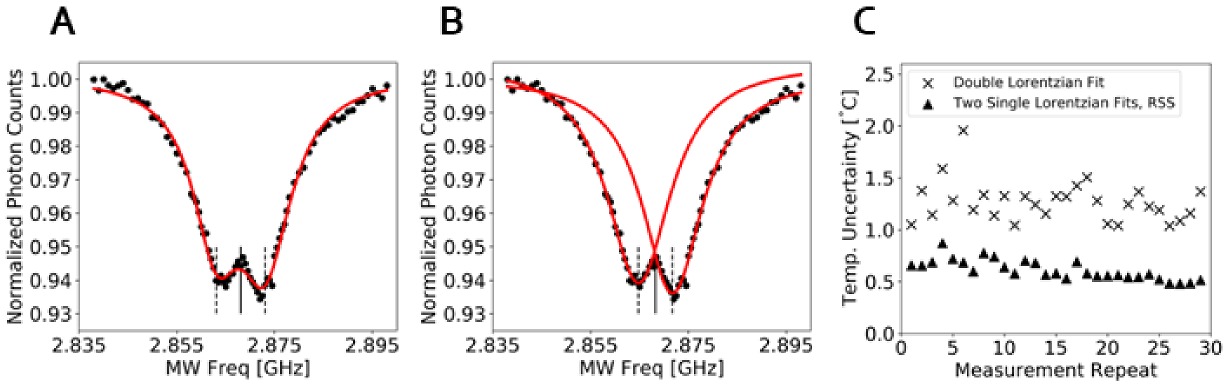

Figure S2. Fitting methods for raw ODMR data (A) Double Lorentzian curve fitting with peak values shown as vertical dotted lines and calculated center frequency shown as a vertical solid line. (B) Two Single Lorentzian curve fitting with peak values show as vertical dotted lines and calculated center frequency shown as a vertical solid line. (C) Temperature uncertainty values calculated from fit uncertainties for 29 consecutive intracellular temperature measurements comparing the Double Lorentzian and Two Single Lorentzian methods.

Temperature calibration was performed for several nano-diamonds to determine their temperature sensitivity, which can vary due to local electric and magnetic fields causing small variations in the NV^- center internal energy levels. Table S1 shows the sensitivity, dD_{gs}/dT, for 4

NV-NDs adhered to a glass slide, determined by measuring the shift in ODMR center frequency while varying the slide temperature with a heater. The glass slide temperature was independently monitored with a platinum RTD temperature sensor. Each point in TableS1(A) represents a measurement from a single ND at a set-point temperature. The measured temperature sensitivity ranges from -89 kHz/°C to -96kHz/°C. A temperature sensitivity of -74kHz/°C has been reported for bulk diamond samples while sensitivity values for NDs are expected to vary due to material strain and surface effects.

Table S1.

A

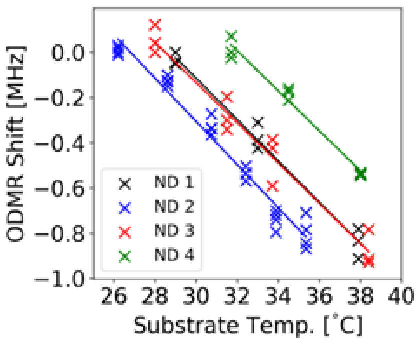

B

ND #	dD_{gs}/dT [kHz/C]
1	-91 ± 5
2	-96 ± 4
3	-89 ± 6
4	-89 ± 5

Table S1. Temperature sensitivity of 4 NV-NDs (A) ODMR center frequency shift for 4 NV-NDs plotted against measured glass slide temperature. (B) Temperature sensitivity and uncertainty values for 4 NV-NDs.

To quantify measurement stability, we generated Allan Deviation plots for NV-ND temperature data collected over a 1 hour period while set point temperature was held constant (Figure S3A). Temperature measurements taken with extracellular NV-NDs (adhered directly to the glass slide) generally have lower noise levels than the intracellular temperature measurements. For intracellular measurements the variability at small time scales (5-10 minutes) is large but drops

below 0.7 °C for averaging times greater than 20 minutes. For this reason, temperature was sampled for a minimum of 20 minutes before and after any perturbation.

We also directly quantified measurement drift by plotting intracellular and extracellular ND based temperature measurements over time in a stable environment (Figure S3B) as well as the incubation chamber temperature as measured by an RTD thermometer in the media (Figure S3C). These measurements were conducted while the ODMR MW excitation and laser illumination were active in order to check for measurement induced heating. The same microwave and laser power was used for all subsequent measurements.

Figure S3.

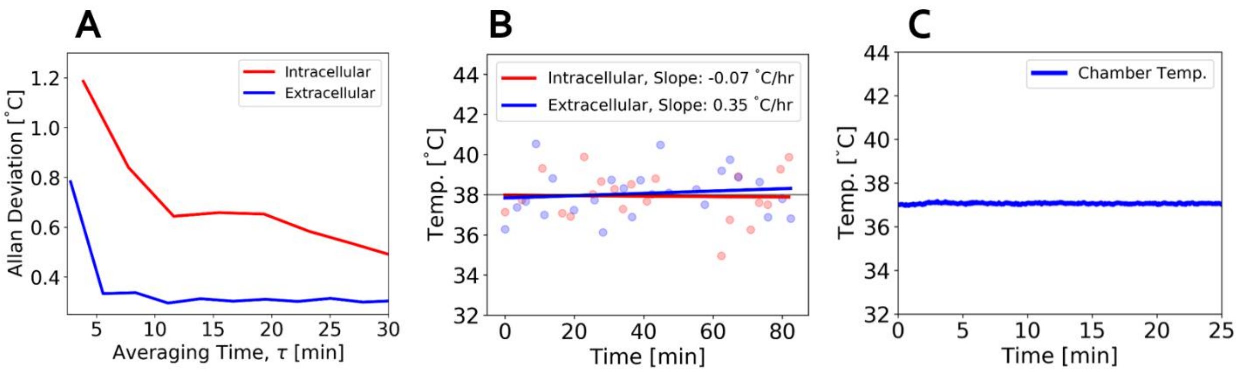

Figure S3. (A) Allan Deviation of temperature measurement collected over a 1-hour period for one Intracellular NV-ND and one Extracellular NV-ND. (B) NV-ND temperature measurement for one Intracellular NV-ND and one Extracellular NV-ND in a stable environment to quantify measurement drift. P-values for Wald Test for a null hypothesis of slope equal to zero: $p_{intracellular}$=0.54, $p_{extracellular}$=0.92 (C) Incubation chamber temperature measured by an RTD thermometer during ODMR MW excitation and laser illumination.

In our work we observed that careful ND selection was critical for a reliable measurement. Several parameters of the ODMR data from each ND were evaluated to select one that is optimal for stable intracellular temperature readings. While preparing for intracellular temperature

measurement it is important to select NV-NDs with ODMR shape parameters that will result in successful Lorentzian fitting. The shape parameters shown in Table S2 were determined empirically as criteria for an NV-ND to be used for temperature measurements. A flow chart is also included to illustrate the process used to identify suitable NDs. Approximately one ND out of ten tested would pass the selection criteria and be used for temperature measurement. In the second step suitable cells were selected based on morphology and proximity to MW wire (<30μm).

Table S2.

Criteria	Value
Brightness	> 1Mc/s
Contrast (Left)	> 3%
Contrast (Right)	> 3%
FWHM (Left)	< 15MHz
FWHM (Right)	< 15MHz
Fitting Uncertainty	< 2 °C

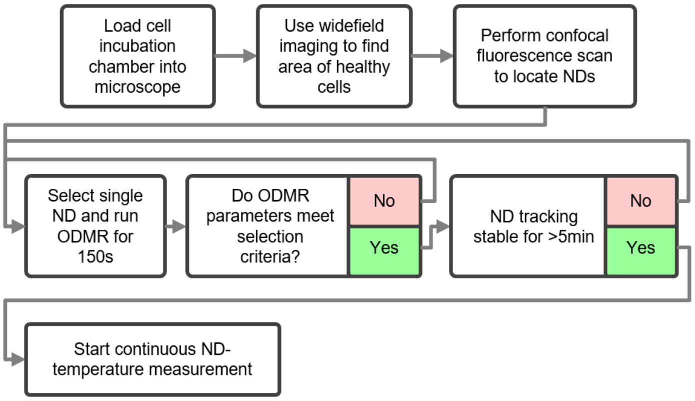

Table S2. Empirically determined ODMR shape parameters used for selecting NV-NDs for temperature measurement and flow chart showing ND selection process followed before starting temperature measurement.

After continuous temperature measurement is initiated each ODMR curve is also assessed for quality. ODMR curves were excluded if the Lorentzian fitting did not meet a threshold on the goodness of fit metric or if the baseline brightness changed significantly during data collection (usually indicating momentary loss of focus due to cell motion).

Localization of intracellular NDs

Imaging performed for intracellular localization of lysosomes and mitochondria was using LysoTracker Green (excitation at 504 nm and emission at 511 nm) and MitoTracker DeepRed (excitation at 644 nm and emission at 665 nm).

In the Supplemental Information video "Video of NanoDiamonds near Mitochondria.AVI" the deep red color indicates the position of intracellular NDs and the gradient of orange shades indicates the position and intensity of the MitoTracker DeepRed fluorescence signal which is normalized to the mean. Each frame of this time-lapse video is separated by six seconds, allowing for visualization of how the cell morphology changes, how the intracellular NDs move, and how mitochondria density changes over time. The video represents a total elapsed time of 3 minutes.

Selecting FCCP Concentration

Towards the goal of selecting an FCCP concentration to stimulate metabolism while maintaining cell viability we measured oxygen consumption rate of a population of U251 cells subject to a range of FCCP concentrations (Figure S4). At concentrations of 1μM or lower, OCR increased sharply in the 10 minutes following administration but then tapered off over the next hour. At concentrations of 2μM and higher the elevated OCR was sustained for longer than 1 hour.

Figure S4.

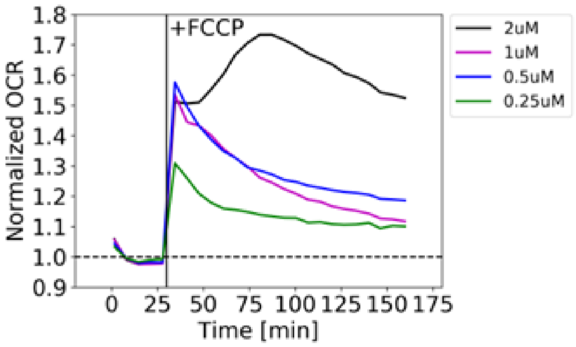

Figure S4. Results of Extracellular Flux measurement of normalized Oxygen Consumption Rate (OCR) as a function of four different concentrations of FCCP for a population of 20,000 U251 cells.

ODMR Shape Parameters during FCCP and Control Experiments

Non-thermal intracellular changes that would result in increased local electric fields or ND surface charges have been suggested as possible confounders and are expected to be associated with significant changes in ODMR full-width at half maximum (FWHM) and asymmetry of ODMR contrast. Because NV-ND temperature measurement relies on quantifying the shift of the ODMR center frequency we want to ensure that this shift is not correlated with changes to the ODMR shape parameters in our experimental results. Comparing the contrast and linewidth parameters of the ODMR spectrum before and after FCCP stimulation (Figure S5) shows that FWHM and contrast do change throughout our experiment but not with a consistent magnitude or direction, and also the ODMR center frequency shift appears independent when considering the four experimental repeats.

These results can be compared to the ODMR shape parameters that were tracked during the control experiments (Figure S6, S7). In the control experiments the contrast and linewidth parameters exhibit similar changes as in the FCCP experiment, but in this case, there is no significant change in the OMDR center frequency shift across the four experimental repeats.

Figure S5.

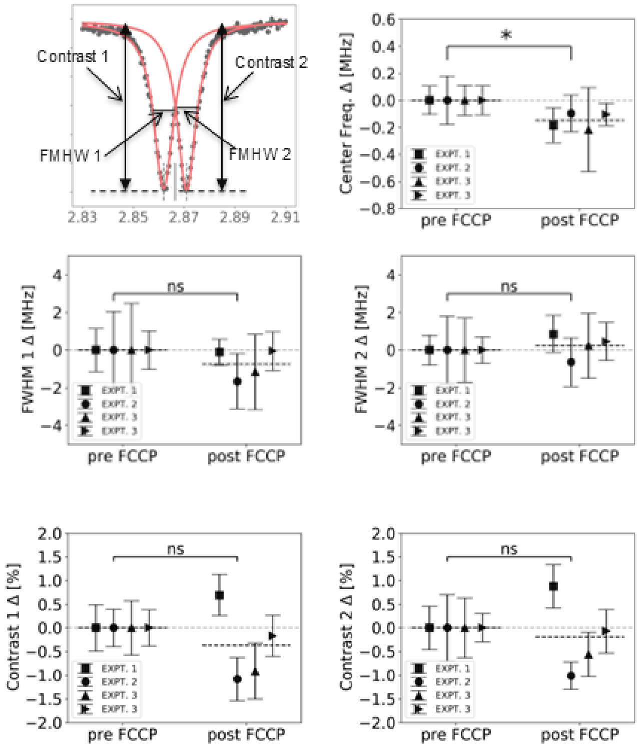

Figure S5. Change in ODMR shape parameters from intracellular NV-NDs before and after administration of 5μM FCCP (n=4).

Figure S6.

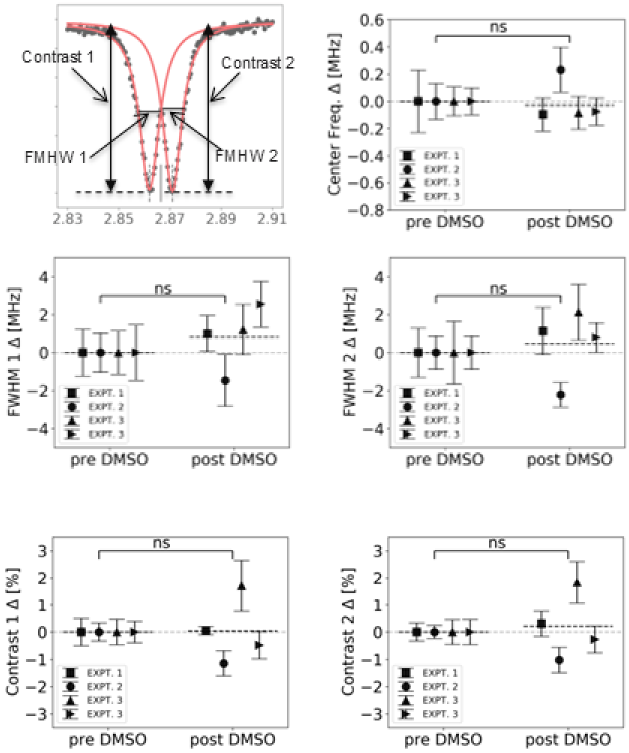

Figure S6. Change in ODMR shape parameters from intracellular NV-NDs before and after administration of 0.125μL DMSO (n=4).

Figure S7.

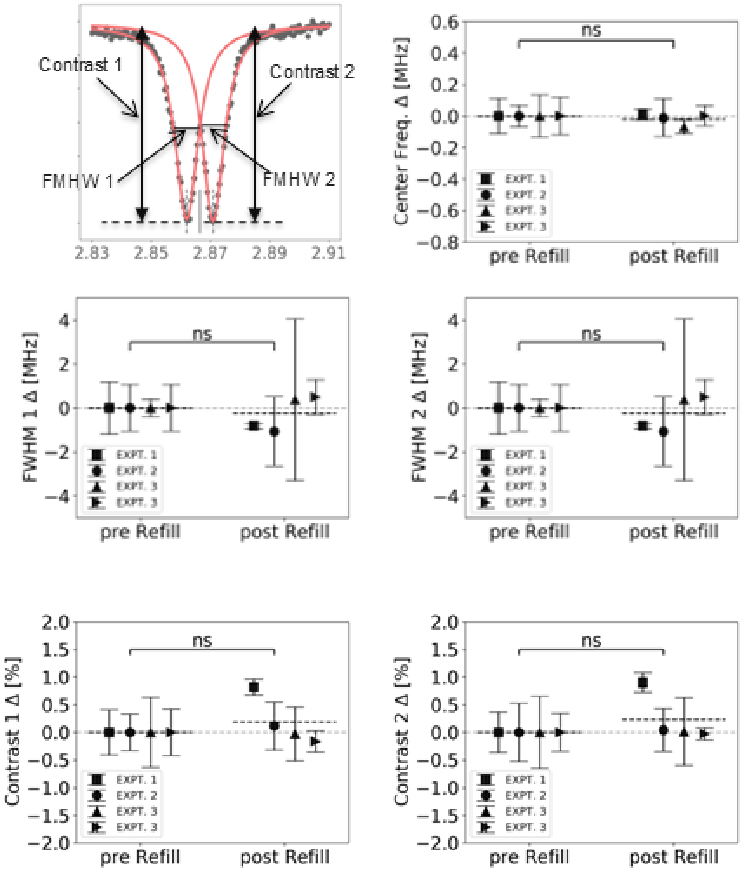

Figure S7. Change in ODMR shape parameters from intracellular NV-NDs before and after refilling incubation chamber media (n=4).

Methods

Custom built microscope

Fluorescent laser illumination was performed with a Laser Quantum GEM 532nm 2W laser attenuated with 2 neutral density filters (1x ThorLabs NE13A, 1x ThorLabs NE10A) to a 100μW laser power (measured with ThorLabs PM100D). Laser light was collimated with a lens (ThorLabs AC254-075-A-ML) and was incident on a Gooch & Housego 3350-120 acousto-optical modulator (AOM), a half-wave plate (ThorLabs WPH10M-53) and then another lens (ThorLabs AC254-150-A-ML) that focused laser light into a fibercoupler (Newport F-916, F-915T). Laser power after the fibercoupler was measured as 15μW. After the fibercoupler laser light was passed through a 530 +/- 21nm filter (ThorLabs MF530-43) and another half wave plate (ThorLabs WPH10M-53). A mirror then reflected the laser into a beamsplitter (Thor Labs BSF20-B) that reflected green (excitation) light and transmitted red (emission) light. The reflected, green, laser light was redirected by a 90 degree mirror into the 60x (NA 1.35) oil-immersion objective (Olympus UPLSAPO60XO) mounted on a custom-made holder coupled to a 3-axis piezo stage (NPoint NPXY200Z25-103) controlled by an NI DAQ USB-6343. The objective was heated with a heating collar (ThorLabs TLK-H Flexible Heater) used to get the cell media to the desired 38 °C temperature. The objective was focused on a custom-made chamber containing cells and media (detailed below) mounted on a manual XYZ stage. Above the chamber was a custom-made tube with a condenser (Thor Labs ACL25416U-A) and a white LED light (ThorLabs ACL25416U-A) for transmission microscopy.

Fluorescence light emitted from the sample was reflected by the 90° mirror into the beam sampler and onto a lens on an xyz stage that focused it into a pinhole (ThorLabs P25S). After the pinhole the emitted light went through a >650nm filter and was collimated by a lens (SPEC) onto

a photon counter (Excelitas SPCM-AQRH-13) mounted on a manual XYZ stage (Thor Labs PT3A).

For wide-field transmission microscopy, a flip mirror (Thor Labs TRF90) was used to direct the white LED light after it was reflected by the 45° mirror. The flip mirror directed light into a lens (Thor Labs AC254-035-A-ML) mounted on a CCD camera (Zelux CS165CU). For fluorescence imaging the mirror was flipped in the vertical direction allowing laser light to pass, and the white LED light was turned off.

For microwave irradiation, a microwave source (Rohde & Schwarz SMIQ03B) was used in conjunction with a 16W amplifier (Mini-Circuits ZHL-16W-43-S+) which connects directly to the SMA port on the cell incubation chamber.

All electronics hardware were controlled via a PC running a user configured version of the open-source Qudi python suite together with a custom Jupyter Notebook running in the Qudi Kernel.

Cell chamber

The cell chamber used to culture cells and measure cell temperature was integrated with a custom circuit board for MW irradiation. A custom circuit board (fiberglass-reinforced epoxy substrate with copper foil ground plane) was fabricated with a square cut out for access to the coverslip, copper conducting strips and two SMA ports (one for MW signal input and one for 50 Ohm termination). A #1.5 coverslip was glued to the center of the circuit board and a gold 20μm microwire (Goodfellow 1000034323) was placed across it. The microwire was soldered to the copper conducting strips at the two ends such that the wire was flush with the glass coverslip. A PDMS chamber (Grace Bio-Labs FlexWell™ Incubation Chambers, 204908) was then attached on top. An RTD sensor (Thor Labs TH100PT) was attached to the corner of the coverslip inside of the PDMS chamber and let dry overnight. The chamber was then washed with deionized water

and 70% ethanol. Once inside of the cell culture hood, the chambers were washed 3 more times with phosphate buffered saline (PBS) and left under UV light for further disinfection for at least 30 minutes. The chamber slides were coated with 400µl of Poly-L-Lysine solution obtained from Millipore Sigma (cat # P4707) for 5 minutes and then washed with PBS 3 times. The chambers were then left to dry for at least 30 minutes. Cells were then added to the chambers as detailed below.

Cell culture

A U251 glioblastoma cell line (A gift from the lab of Dr. Adrienne A. Boire, verified via STR by Dr. Elizabeth Coffee) was cultured in standard growth media (DMEM HG, 10% FBS, PS). The night before scheduled cell experiments, cells were split and counted. Approximately 2.5×10^5 cells were added to each chamber after the chambers were left to dry, as described above. Overall, about 1mL of media was added to the chamber with the cells. The cells were left to attach for at least 3 hours. After the allotted attachment time, 80uL of the nanodiamond suspension (detailed below) were added to the cells and left to incubate overnight. In the morning, before experiments, cell media from our chamber was replaced with fresh growth media and 25µM of HEPES were added. The chamber was then transferred to a preheated microscope.

Preparing NDs

A custom-made nanodiamond stock suspension was initially fabricated by Pureon AG, and subsequently electron-irradiated and annealed at the Leibniz Institute of Surface Engineering. Electron-irradiation was performed at high temperature (800 °C) with an irradiation dose of 0.75×10^{18} cm^{-2} and subsequent air-oxidization at 620 °C. Resulting nano-diamonds have an average NV density of 1.5 ppm and an average particle size of 100 nm resulting in an average of 140 NVs per ND.

The stock solution of nanodiamonds was sonicated for 30 minutes on Fisher Sonicator (CPX 2800 Ultrasonic Bath 2.8L). NDs were then diluted: 5µl of ND stock suspension was diluted with 500µl of media. The diluted suspension in media was then sonicated for another 90min. After the long sonication, 80µL of the diluted ND suspension was added to adherent cells as explained above. A stock ND suspension was diluted in cell media and sonicated as detailed in the Methods. A cell plate at 80% confluence was then incubated with the ND suspension overnight.

Imaging and sampling temperature

After culturing cells and implanting NDs the cell incubation chamber was transferred to our custom dual path microscope which has been pre-heated to 38°C. The microscope was first used in the widefield imaging mode, with the CCD camera and an illuminated backlight. We assessed cell health by observing morphology and chose an area of healthy monolayer cells within 30µm of the MW wire. We then rotated the flip mirror to switch to the confocal imaging path, partially dimmed the backlight and performed a confocal scan using the Qudi Software. This scan created an image that showed cell outlines as well as the fluorescent NDs.

We selected an ND that was separated from others (not aggregated with other NDs) and that had a brightness greater than 1Mcounts/s. We then used the Qudi POI manager to track the selected ND by refocusing in 10 second intervals. Tracking and observing the ND motion over 5 to 10 minutes confirmed intracellular activity. After successful ND tracking, we initiated temperature measurement controlled via a Jupyter Notebook that runs on the Qudi Python Kernel. The temperature measurement notebook alternates between 10 seconds of collecting ODMR data and 10 seconds of ND tracking for a total elapsed time of 150 seconds for each temperature measurement point.

This temperature sampling process was run continuously during a temperature measurement experiment except during addition of media or administration of a drug to the cell incubation chamber in which case we temporarily paused temperature measurement and changed to a tracking-only mode. This was done as a precaution to continue tracking the selected ND while the media or drug was manually introduced into the open area in the top of the cell incubation chamber. By following this method we ensure that the same ND is used throughout the course of the entire experiment.

Data Analysis

After collecting ODMR data during a temperature measurement experiment we performed data analysis using a Jupyter Notebook which imported the raw ODMR data, performed curve fitting as described in Figure S1, and plotted temperature versus time for the duration of the experiment. Our analysis program discarded temperature points if the ODMR curve fitting resulted in an uncertainty greater than 2°C or if the ND brightness changed by more than 10% during measurement (usually indicating loss of focus due to ND motion).

Our program also plotted and saved ODMR shape parameters for each temperature point including brightness, contrast, splitting and FWHM for both ODMR peaks.
